# Health literacy in an urban elderly East-German population – results from the population-based CARLA study

**DOI:** 10.1186/s12889-015-2210-7

**Published:** 2015-09-10

**Authors:** Daniel Tiller, Beatrice Herzog, Alexander Kluttig, Johannes Haerting

**Affiliations:** Institute of Medical Epidemiology, Biostatistics, and Informatics, Martin Luther University of Halle-Wittenberg, Magdeburger Straße 8, Halle (Saale), Germany

## Abstract

**Background:**

Health literacy (HL) has gained increasing attention in public health research. However, until now research was mainly focused on clinical settings rather than on the general population. Due its relation to social determinants and health outcomes, HL is of special interest in epidemiological studies. The aim of the present study was therefore to describe HL among an elderly general high-risk population, to analyze the potential contributing factors of HL, and to analyze the impact of HL on health-related outcomes**.**

**Methods:**

We used data from the CARLA Study, which is a prospective population-based cohort study of the elderly general population of the city of Halle (Saale) in Eastern Germany. The short version of the HLS-EU Questionnaire (HLS-EU-Q16) was administered with 1,107 subjects aged between 55 and 91 year old. A HL score ranging from 0 to 50 points was computed and classified according to the recommendation of the HLS-EU project. Socio-economic as well as health-related variables were determined during the standardized interview and clinical examination. We calculated linear as well as logistic regression models in order to analyze the association between HL and health-related outcomes as well as potential influencing factors of HL.

**Results:**

Overall, the HL score was 36.9 (SD 6.9). Among all subjects, 4 % showed inadequate HL, 23 % problematic HL, 50 % sufficient HL, and 23 % excellent HL. HL was positively associated with educational level, net household income, and self-perceived social position. Further, we found an increase of HL with age (β = 0.10; 95 % CL 0.05; 0.15) and a lower HL score among women compared with men (Diff = -1.4; 95 % CL −2.2; −0.6). An inverse association was observed between HL and diabetes among both sexes (OR 0.93; 95 % CL 0.93; 0.98), between HL and myocardial infarction among women, and between HL and stroke among men.

**Conclusions:**

In this elderly general Eastern German population, we found higher HL score values compared with previous studies using the same questionnaire. HL was associated with socio-economic status. Furthermore, this cross-sectional study could show associations between HL and different health-related outcomes even after adjustment for educational level. However, further research is needed in order to evaluate the impact of HL on health-related outcomes using longitudinal data derived from the general population.

## Background

In recent decades the concept of health literacy (HL) has gained increasing attention in public health research. HL is considered to be crucial in mediating the impact of social factors and determinants on one’s individual health [1].

There are different concepts of HL, from the simple understanding of health information, such as a physician’s instruction of taking medication, to a comprehensive meaning of HL. The latter approach defines HL as the knowledge and competence to access, understand, appraise, and apply health information for health judgment. This conceptual model of HL integrates three health relevant areas: health care, disease prevention, and health promotion [2].

The concept of HL is closely related to social determinants, health behavior, and health outcomes as well as to the use of health services. Low HL is associated with different health outcomes such as self-perceived health status, mortality, and the use of health care facilities [3, 4]. Therefore, HL is of increasing interest in epidemiological studies.

There are different tools for measuring HL in the population. The most frequently used are the Test of Functional Health Literacy in Adults (TOFHLA) [5] and the Newest Vital Sign Test [6], which measure functional HL, or the Rapid Estimate of Adult Literacy in Medicine (REALM) [7], which assesses HL skills. However, the mentioned assessment tools were developed and mainly applied in clinical settings, and therefore there is only a small amount of research investigating HL in population-based studies using random samples from the general population. In the framework of the European Health Literacy Survey (HLS-EU) a research consortium with members from different European countries developed a questionnaire to measure HL in the general population [8]. This tool is based on the comprehensive approach of HL described above [2].

HL is believed to play a vital role in the risk of development of chronic diseases and their mediation, especially of diseases which are closely related to social factors [1, 9, 10]. Chronic diseases require a large portion of individual health decisions and therefore HL can be an important contributing factor for those conditions. We would expect that HL influences chronic conditions presuming that lower HL leads to higher risk of chronic diseases and vice versa. However, there are sparse data describing the relationship between HL and health-related variables such as chronic diseases as well as health-related quality of life (HRQL) especially using large random samples from the general population [3, 11]. Furthermore, HL is still not well described in high-risk populations. Our cohort consists of an elderly population with an extraordinarily high prevalence of hypertension, diabetes, obesity, and other cardiovascular risk factors compared with other German regions [12, 13]. It can therefore be characterized as a high-risk population.

The aim of the present study was therefore to describe HL among a random sample of the general high-risk population using a short version of the HLS-EU Questionnaire (HLS-EU-Q16) [8] to analyze potential determinants of HL such as socio-economic status and finally to analyze the impact of HL on health-related outcomes such as disease prevalence and HRQL.

## Methods

### Study population

CARLA (cardiovascular disease, living, and ageing in Halle) is a population-based cohort study in Halle (Saale) in eastern Germany. For the baseline investigation, 1,779 participants aged between 45 and 83 years old were recruited between July 2002 and January 2006. A multi-step recruitment strategy aimed to achieve a high response rate. The final response proportion after subtracting exclusions (individuals who passed away prior to the invitation, had moved away, or were unable to participate due to illness) was 64 %. A more detailed description of the CARLA design and the examinations has been described elsewhere [14, 15]. The first follow-up examination for 1,436 participants was done between March 2007 and March 2010 (mean follow-up of 4 years). The second follow-up was conducted between January 2013 and October 2013 and included 1,140 participants.

The study was in accordance with the declaration of Helsinki. All participants gave their written informed consent. The study was approved by the local ethics commission of the Medical Faculty of the Martin Luther University of Halle-Wittenberg.

### Health literacy (HL) assessment

During the second follow-up of the CARLA study in 2013, a German version of the HLS-EU-Q16 was administered with 1,107 subjects aged between 55 and 91 years old during the standardized interview. The HL score for the questionnaires was calculated according to the recommendations of the European Health Literacy Project. Index score was only computed for general HL comprising at least 80 % answered items. In order to compare our results with previous studies we calculated the HL score according to the following formula [16]:

$$ Index=\left( mean\left( per\kern0.5em  Item\right)-1\right)\ast \frac{50}{3} $$

Thus, the final score has a minimum of 0 and a maximum of 50 points. Furthermore, we categorized the HL score according to the threshold values published by the EU consortium as follows: 0–25 ‘inadequate,’ >25–33 ‘problematic,’ >33–42 ‘sufficient,’ and >42–50 ‘excellent’ HL.

### Socio-economic variables

Education, net household income, and type of health insurance were determined in the standardized interview. Subjects were further asked about their self-perceived social status using the MacArthur Scale of Subjective Social Status [17]. Education was classified according to the International Standard Classification of Education as total years of formal education, combining school and vocational training. The educational level was classified as follows: a low level of education (max. secondary school without vocational training), a medium level of education (secondary school with vocational training), and a high level of education (any higher level of education).

The income profile of the participants was classified into three categories of income which approximately define tertiles of the population. The net household income (per month) categories for the CARLA study were therefore classified as follows: low income < €1,500; medium income ≥ €1,500 to < €2,000; high income ≥ €2,000.

### Health-related variables

A physician confirmed cardiovascular diseases (myocardial infarction, stroke) and risk factors were assessed during the personal interview and clinical examination. Furthermore, during the personal interview the participants were asked about their smoking habits and alcohol drinking habits.

Hypertension was defined as mean systolic blood pressure (SBP) equal or greater than 140 mmHg, and/or mean diastolic blood pressure (DBP) equal or greater than 90 mmHg, and/or use of antihypertensive medication according to the ATC code, given the participant had known hypertension. Hypertensive participants were categorized into one of the following four subgroups: (1) unaware of hypertension; (2) aware of hypertension, but not treated with antihypertensive medication; (3) aware of hypertension and treated, but not reaching blood pressure values below 140 mmHg and 90 mmHg; (4) aware of hypertension and treated, reaching blood pressure values below 140 mmHg and 90 mmHg. For the analyses hypertension was further categorized in two dichotomous variables: aware of hypertension vs. unaware of hypertension, and treated hypertension vs. untreated hypertension. Diabetes mellitus (DM) was defined as self-reported physician-diagnosed diabetes and/or use of anti-diabetic medication according to the ATC code. Body mass index (BMI) was calculated as weight in kg divided by height in meters squared. HRQL was determined by the self-administered questionnaire SF-12 [18].

### Statistical analyses

General descriptive statistics were calculated for socio-demographic and health-related variables. Continuous variables were displayed as means with their standard deviation. Categorical variables were displayed as numbers and percentages. To analyze the association between HL and potential influencing factors (such as education, household income, etc.) we calculated linear regression models using the HL score as a dependent variable. On the other hand, to analyze the association between HL and health-related variables (such as myocardial infarction, blood pressure, etc.) we calculated logistic as well as linear regression models using the HL score as an independent variable and the health-related variables as dependent variables. Adjustment for covariates is indicated in the results section. The regression coefficient beta resulting from linear regression as well as the odds ratio (OR) resulting from logistic regression were displayed with their 95 % confidence limits (95 % CL). The internal consistency of the questionnaire was evaluated calculating Cronbach’s alpha [19]. All analyses were done using SAS®, Version 9.4 (SAS Inc., Cary, NC, USA).

## Results

In total, 1,107 subjects (53 % males) could be included in the analysis. The mean age of the subjects was 69.9 (SD 6.7) years. Demographic, socio-economic, and health-related characteristics are shown in Table 1. Two thirds of the study population had an intermediate education and about one half of the study population had a monthly household income between €750 and €1,500. Of all the study participants, 71 (6.4 %) had a prior myocardial infarction and 48 (4.3 %) a prior stroke. Almost 80 % of the study population had hypertension and 210 (19 %) subjects had physician-diagnosed diabetes. Of all the subjects with hypertension, 11.3 % were not aware of their hypertension and 15.2 % of all the subjects with high blood pressure did not take any antihypertensive medication.

**Table 1:** Baseline characteristics of the CARLA study population

	*Male*		*Female*	
	N	Proportion/Mean (SD)	N	Proportion/Mean (SD)
Sociodemographic variables				
Age (yrs)	585	70.1 (9.3)	522	69.5 (8.6)
Educational level				
*Low education*	11	1.9 %	44	8.9 %
*Intermediate education*	324	56.5 %	335	67.7 %
*High education*	238	41.5 %	116	23.4 %
Self-perceived social position				
Scale from 1 to 10	541	5.6 (1.7)	478	5.4 (1.8)
Household income				
*Low (<€1,500)*	143	24.9 %	227	45.3 %
*Intermediate (≥€1,500 to < €2,000)*	162	28.2 %	124	24.8 %
*High (≥€2,000)*	269	46.9 %	150	29.9 %
Type of insurance				
*Statutory health insurance*	549	94.3 %	506	97.5 %
*Private health insurance*	33	5.7 %	13	2.5 %
Health-related variables				
Self-perceived general health situation				
*Excellent*	8	1.4 %	6	1.2 %
*Very good*	91	15.6 %	67	12.9 %
*Good*	353	60.6 %	296	57.0 %
*Fair*	108	18.5 %	130	25.1 %
*Poor*	23	4.0 %	20	3.9 %
SF-12 Physical Health Scores	565	46.5 (9.2)	496	44.3 (10.1)
SF-12 Mental Health Scores	565	51.8 (10.2)	496	49.1 (11.4)
SBP (mmHg)	549	128.7 (18.7)	466	124.7 (19.5)
DBP (mmHg)	549	74.4 (10.8)	466	73.5 (9.8)
Body mass index (BMI) (kg/m^2^)	547	28.7 (4.1)	467	29.2 (5.4)
Medical consultation last quarter				
*Never*	45	8.1 %	35	6.9 %
*1–2*	243	43.9 %	230	45.6 %
*3–4*	163	29.4 %	155	30.8 %
*>4*	103	18.6 %	84	16.7 %
Disease prevalence:				
Myocardial infarction	60	10.3 %	11	2.1 %
Stroke	31	5.3 %	17	3.3 %
Hypertension	434	79.5 %	359	77.0 %
Diabetes mellitus (DM)	120	20.5 %	90	17.2 %

### Analysis of the HLS-EU-Q16 single items

Cronbach’s alpha for the HLS-EU-Q16 questionnaire was 0.88. The answers for the single items are shown in Table 2. Almost all subjects indicated that it is easy (30 %) or very easy (68 %) to understand instructions from a general practitioner or pharmacist on how to take prescribed medicine (Q8 of the HLS-EU-Q16). Furthermore, most subjects indicated that it is easy or very easy (97 %) to understand health warnings about behavior such as smoking, low physical activity, and drinking (Q21) or to understand the need for health screenings (Q23). For 44 % of all subjects it is difficult to trust the information on health risks provided by public media (Q28). Only 13 % rated this topic as easy. On the other hand, 45 % of the subjects declared to have difficulties with this topic. For 35 % of the study population it is difficult to decide if a second opinion from another physician is necessary to make an appropriate health decision (Q11).

**Table 2:** Number and percentage of HLS-EU-Q16 items for the study population (*n* = 1,107)

Item	*On a scale from very easy to very difficult, how easy would you say it is to: …*	Very difficult	Fairly difficult	Fairly easy	Very easy	Miss
Q2	…find information on treatments of illnesses that concern you?	28 (2.5 %)	137 (12.4 %)	566 (51.1 %)	370 (33.4 %)	6 (0.5 %)
Q4	…find out where to get professional help when you are ill?	21 (1.9 %)	92 (8.3 %)	493 (44.5 %)	490 (44.3 %)	11 (1.0 %)
Q5	…understand what your doctor says to you?	13 (1.2 %)	107 (9.7 %)	615 (55.6 %)	361 (32.6 %)	11 (1.0 %)
Q8	…understand your doctor’s or pharmacist’s instruction on how to take prescribed medicine?	2 (0.2 %)	25 (2.3 %)	327 (29.5 %)	741 (66.9 %)	12 (1.1 %)
Q11	…judge when you may need to get a second opinion from another doctor?	49 (4.4 %)	336 (30.4 %)	460 (41.6 %)	234 (21.1 %)	28 (2.5 %)
Q13	…use information the doctor gives you to make decisions about your illness?	13 (1.2 %)	194 (17.5 %)	579 (52.3 %)	304 (27.5 %)	17 (1.5 %)
Q16	…follow instructions from your doctor or pharmacist?	2 (0.2 %)	25 (2.3 %)	426 (38.5 %)	640 (57.8 %)	14 (1.3 %)
Q18	…find information on how to manage mental health problems like stress or depression?	50 (4.5 %)	269 (24.3 %)	478 (43.2 %)	276 (24.9 %)	34 (3.0 %)
Q21	…understand health warnings about behavior such as smoking, low physical activity and drinking too much?	4 (0.4 %)	32 (2.9 %)	319 (28.8 %)	735 (66.4 %)	17 (1.5 %)
Q23	…understand why you need health screenings?	5 (0.5 %)	21 (1.9 %)	306 (27.6 %)	760 (68.7 %)	15 (1.4 %)
Q28	…judge if the information on health risks in the media is reliable?	47 (4.3 %)	443 (40.0 %)	451 (40.7 %)	146 (13.2 %)	20 (1.8 %)
Q31	…decide how you can protect yourself from illness based on information in the media?	33 (3.0 %)	293 (26.5 %)	531 (48.0 %)	226 (20.4 %)	24 (2.2 %)
Q33	…find out about activities that are good for your mental well-being?	10 (0.9 %)	128 (11.6 %)	561 (50.7 %)	384 (34.7 %)	24 (2.2 %)
Q37	…understand advice on health from family members or friends?	23 (2.1 %)	153 (13.8 %)	516 (46.6 %)	391 (35.3 %)	24 (2.2 %)
Q39	…understand information in the media on how to get healthier?	16 (1.5 %)	206 (18.6 %)	577 (52.1 %)	285 (25.8 %)	23 (2.1 %)
Q43	…judge which everyday behavior is related to your health?	6 (0.5 %)	58 (5.2 %)	522 (47.2 %)	500 (45.2 %)	21 (1.9 %)

### Health literacy (HL) score and socio-demographic factors

We could calculate the HL score in 1,033 subjects. Overall, the mean of the HL score was 36.9 (SD 6.9). According to the above-mentioned classification of the HL score among all subjects, 4 % showed inadequate HL, 23 % showed problematic HL, 50 % showed sufficient HL, and 23 % showed excellent HL.

The mean HL score was 37.6 (SD 6.6) for men and 36.2 (SD 7.2) for women. HL score was 1.4 points lower among women compared with men (95 % CL −2.2; −0.6). HL increased among men aged under 60 years from 36.1 (SD 6.8) to 39.0 (SD 6.2) among men aged over 80 years. In women, the HL score increased from 35.1 (SD 7.8) among age groups under 60 years to 37.5 (SD 8.5) among age groups over 80 years. HL score increased per year by 0.12 (95 % CL 0.06; 0.18) among men and 0.06 (95 % CL −0.01; 0.14) among women. HL score was related to education. Male subjects in the group with the highest education level had a higher score by 7.4 points (95 % CL 3.3; 11.4) compared with male subjects in the lowest education group. Among women association was slightly lower. Women with the highest level of education had a higher HL score by 3.8 points (95 % CL 1.2; 6.5). Furthermore, HL was associated with self-perceived social position, HL score increased by 0.7 points (95 % CL 0.3; 1.0) per point of the MacArthur scale of social status in men and by 0.8 points (95 % CL 0.4; 1.2) in women. We observed in our study that subjects with private health insurance had a slightly higher HL score (37.4 (SD 6.5)) than subjects with statutory health insurance (36.9 (SD 6.9)). Further results of the association between socio-demographic factors and HL are shown in Tables 3 and 4.

**Table 3:** HL score and categories by sex, age, level of education and type of insurance

		Number (%)				Mean (SD)
	*HL Score Categories*	Inadequate (0–25)	Problematic (>25–33)	Sufficient (>33–42)	Excellent (>42)	HL Score
Total		45 (4.4 %)	234 (22.7 %)	519 (50.2 %)	235 (22.8 %)	36.9 (6.9)
Sex	Male	18 (3.3 %)	108 (19.6 %)	286 (52.0 %)	138 (25.1 %)	37.6 (6.6)
	Female	27 (5.6 %)	126 (26.1 %)	233 (48.2 %)	97 (20.1 %)	36.2 (7.1)
Age	<60 years	12 (7.6 %)	38 (24.2 %)	78 (49.7 %)	29 (18.5 %)	35.6 (7.2)
	60 to <70 years	13 (3.4 %)	104 (27.3 %)	186 (48.8 %)	78 (20.5 %)	36.5 (6.5)
	70 to <80 years	16 (4.6 %)	67 (19.4 %)	180 (52.0 %)	83 (24.0 %)	37.4 (6.9)
	≥80 years	4 (2.7 %)	25 (16.8 %)	75 (50.3 %)	45 (30.2 %)	38.3 (7.3)
Level of education	Low	11 (22.9 %)	11 (22.9 %)	21 (43.8)	5 (10.4 %)	32.4 (9.2)
	Medium	22 (3.6)	138 (22.4 %)	308 (50.0 %)	148 (24.0 %)	37.2 (6.7)
	High	12 (3.6 %)	76 (22.6 %)	171 (50.9 %)	77 (22.9 %)	37.2 (6.7)
Health insurance	Statutory	43 (4.4 %)	224 (22.7 %)	496 (50.4 %)	222 (22.5 %)	36.9 (6.9)
	Private	2 (4.6 %)	9 (20.5 %)	22 (50.0 %)	11 (25.0 %)	37.4 (6.5)

**Table 4:** Association between and socio-demographic variables and HL^a^

	*Male*		*Female*	
	β	95 % CL	β	95 % CL
Age (years)	0.12	0.06; 0.18	0.06	−0.01; 0.14
Educational level^a^				
*Low education*	−7.36	−11.44; −3.29	−3.83	−6.50; −1.17
*Intermediate education*	0.46	−0.64; 1.56	−0.15	−1.69; 1.40
*High education*	-	-	-	-
Self-perceived social position^b^				
*Scale from 1 to 10*	0.70	0.34; 1.00	0.80	0.42; 1.17
Household income^b^				
*Low (<€1,500)*	−1.68	−3.15; −0.21	−1.28	−2.96; 0.39
*Intermediate (≥€1,500 to < €2,000)*	−0.29	−1.65; 1.06	0.16	−1.67; 1.99
*High (≥€2,000)*	-	-	-	-

^**a**^adjusted for age

^**b**^adjusted for age and education

### Health literacy (HL) score and health-related outcomes

The results of the association between health-related variables and HL are shown in Table 5. We did not observe an association between blood pressure and HL nor between BMI and HL.

**Table 5:** Association between HL and health-related outcomes^a^

	Male		Female	
	β	95 % CL	β	95 % CL
Health-related variables				
Systolic blood pressure (SBP) (mmHg)	0.12	−0.13; 0.37	−0.02	−0.29; .024
Diastolic blood pressure (DBP) (mmHg)	0.07	−0.06; 0.21	−0.10	−0.23; 0.03
Body mass index (BMI) (kg/m^2^)	−0.01	−0.06; 0.05	0.02	−0.06; 0.09
Health-related quality of life (HRQL)				
*SF-12 Physical Health Scores*	0.26	0.14; 0.37	0.31	0.20; 0.43
*SF-12 Mental Health Scores*	0.34	0.21; 0.47	0.52	0.38; 0.65
Number of medical consultations in the last quarter	−0.05	−0.10; −0.01	−0.02	−0.04; 0.01
	OR	95 % CL	OR	95 % CL
Disease prevalence:				
*Myocardial infarction*	1.00	0.95; 1.04	0.94	0.87; 1.02
*Stroke*	0.91	0.85; 0.97	1.06	0.99; 1.15
*Hypertension*	0.99	0.95; 1.02	1.00	0.97; 1.04
*Diabetes mellitus (DM)*	0.96	0.93; 0.99	0.93	0.90; 0.97

^**a**^adjusted for age and education

A higher HL score was associated with a lower chance of DM in men (OR = 0.96; 95 % CL 0.93; 0.99) as well as in women (OR 0.93; 95 % CL 0.90; 0.97). Overall, an increase of the HL score by one point was associated with a decrease of the odds of having diabetes by a relative 0.05 (OR 0.95; 95 % CL 0.93; 0.98). For stroke we found a similar association only in men (OR 0.91; 95 % CL 0.85; 0.97). The effect in women was opposite (OR 1.06; 95 % CL 0.99; 1.15). HL was not associated with hypertension. In women we could see an effect with myocardial infarction (OR 0.94; 95 % CL 0.87; 1.02).

HL was associated with HRQL. For men, a physical health score of HRQL increased by 0.26 (95 % CL 0.14; 0.37) and by 0.31 (95 % CL 0.20; 0.43) in women. The increase was even higher for the mental health score of HRQL with 0.34 (95 % CL 0.21; 0.47) among men and 0.52 (95 % CL 0.38; 0.65) among women.

We could not identify an association between treatment of hypertension and HL. However we found a weak association between awareness of hypertension and the HL score among women. HL was negatively associated with the use of health care facilities measured by the number of consultations in the last three months (β –0.03; 95 % CL −0.06; −0.01).

## Discussion

**Fig. 1:**
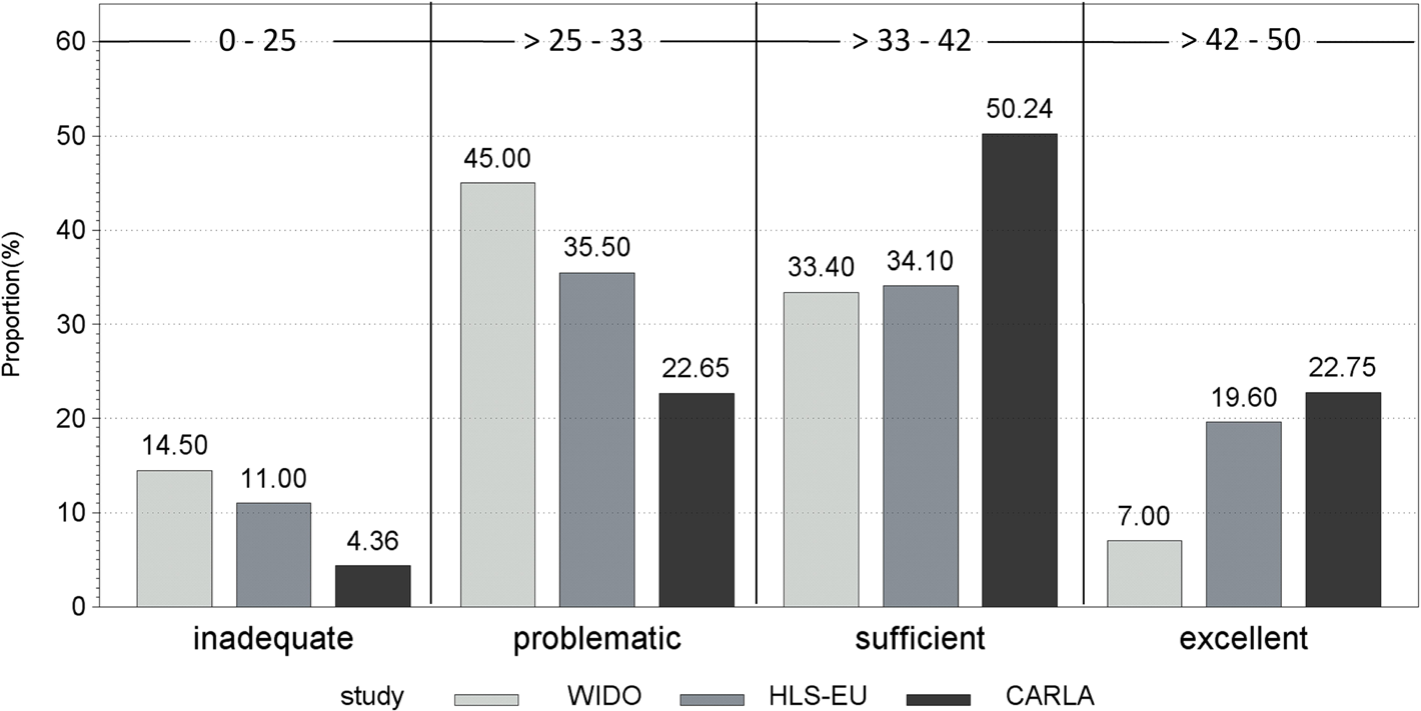
Comparison of health literacy (HL) between three German studies

We aimed to describe HL among an Eastern German, urban and elderly population using a comprehensive measurement tool developed by the HLS-EU project. We found higher HL in our study population compared with previous studies using the same questionnaire. A representative survey of HL among users of the statutory health insurance (SHI) in Germany revealed a considerably lower HL (WIDO study) [20]. The HL score therein was 31.9 compared with 36.9 in our study population. Comparing our results with the German sample of the HLS-EU project (HL score 34.5 (SD 7.9)), our study population shows higher HL as well [8]. However, the age range (for the WIDO study 18 years and more and for the HLS-EU project 15 years and more) differs substantially between our study population and those of the two mentioned studies and at least in CARLA the HL score increases with age. Figure 1 shows a comparison between our data and the aforementioned two studies regarding the HL categories. Only 7 % of the subjects of the WIDO study have excellent HL compared with 22 % of the subjects in our study. Of all the participating countries in Europe, 17 % of the subjects showed excellent HL. HL was highest in the Netherlands and lowest in Bulgaria [8].

We observed an increase of HL with age. This observation is in line with the aforementioned WIDO study. In this study, the HL score was lowest for subjects aged 30 to 40 years and highest among subjects aged over 65 years [20]. A recently published study from Japan showed an increase of HL with age as well [21]. However, in the European Study HL declined with age in all countries except for the Netherlands. An additional analysis of the Dutch study population showed that the association of HL and age varies between different subdomains [22]. Further prior studies showed a decrease of HL with age, as well [4, 23–26]. However, these studies used different tools to measure HL, mainly TOFHLA. The explanation for these unexpected results could be the fact that CARLA especially comprises the older population and older people are more concerned with health-related topics due to the increasing risk of chronic diseases in older age. Furthermore, HL in the way it is measured may increase according to the necessity to deal with one’s own health problems. The above-mentioned tools, such as TOFHLA or REALM, are mainly focused on patient’s skills, which may decrease with age. In contrast, HLS-EU-Q16 represents one’s self-assessed ability to deal with health problems, which may increase with age.

In our study population, women had lower HL than men. Results from prior studies regarding sex-specific differences are inconclusive. A meta-analysis from 2004 conducted in the United States concluded that there are no differences in HL between men and women [27]. However, German studies using the same questionnaire as that used in our study showed that women have better HL than men [8, 20]. A study conducted in Albania using the HLS-EU-Q47 did not show a difference between men and women regarding HL score, either [28].

We found an association between HL and education as well as with self-perceived social status. Subjects with low education had significantly lower HL scores than subjects with the highest educational level. Furthermore, HL score increased the higher one’s self-perceived social status was. Both educational level and social status have been described as being associated with HL in prior studies [20, 22, 25]. The above-mentioned Dutch study [22] pointed out that educational level seems to mainly affect the dimension “accessing and understanding health information” and to a lesser extent the dimension “appraising and applying health information.” However, we could not analyze these associations due to the 16-item short questionnaire used in our study.

Analyzing the single items according to the dimension “accessing, understanding, appraising and applying health information” revealed that for our subjects have the least difficulty with the dimension “understanding health information” and highest difficulty with the dimension “appraising health information”. Subjects seem to have the most trust in general practitioners and pharmacists and less trust in information provided by public media.

### Health-related outcomes

In our study, we did not find an association between HL and blood pressure as an outcome variable. Prior studies did not reveal such an association, either [20, 29]. In our study population HL was furthermore not associated with BMI. While the German WIDO study also failed to show such an association, a study conducted in Portugal using HLS-EU-Q47 showed an association between HLS and BMI [30]. However, the results from this last study were drawn from a convenience sample which limits their interpretation.

We could identify an association between HL as an exposure variable and stroke among men. A higher HL score was associated with lower odds of having had a history of stroke. Furthermore, we could show an association between HL and DM for both sexes. While the German WIDO study could not identify an association between HL and chronic diseases such as diabetes, hypertension, or coronary heart disease, other studies did show an association with chronic diseases such as diabetes [3, 31, 32]. Our cross-sectional approach does not allow some causal interpretations to be made, as we do not know the direction of the association. Moreover, the mechanism between HL and chronic diseases cannot be explained with this approach. However, the observed association between HL and diabetes leads to the assumption that parts of social factors can be explained with the concept of HL.

HL was clearly associated with self-perceived HRQL. Both physical health scores and mental health score derived from the SF-12 were positively associated with the HL score. These results are confirmed by other studies such as the HLS-EU study [8, 20].

### Strengths and limitations

To our knowledge, this is the first study comprehensively analyzing HL with the HLS-EU-Q16 in a random representative sample of the general population. Nevertheless, some limitations need to be recognized. Due to the fact that we analyzed data from the second follow-up of the CARLA study, the representativeness of our study population could be questionable due to loss to follow-up. Therefore, we repeated all analyses using drop-out weights for each participant derived from logistic regression models with loss to follow-up as an outcome variable. However, the results from these sensitivity analyses did not differ from the results of the primary analyses. Therefore, we only present the unweighted results.

Our study population comprises subjects aged over 55 years. Furthermore, several previous studies used different questionnaires to measure HL. Therefore, the comparison with other studies is limited. Furthermore, due to the cross-sectional nature of the data, caution must be exercised in the interpretation of the results, especially concerning the association between HL and potential health-related outcomes. Regarding the study population, we cannot rule out the possibility of a selection bias.

### Conclusion

We found higher HL compared with previous studies. HL was associated with levels of education, household income, and with self-perceived social position. Furthermore, this cross-sectional study could show associations between HL and different health-related outcomes even after adjustment for educational level. However, further research is needed in order to evaluate the impact of HL on health-related outcomes using longitudinal data derived from the general population.

## Abbreviations

95 % CL: 95 % confidence limits
ATC code: Anatomic Therapeutic Classification
BMI: body mass index
CARLA: Cardiovascular Disease, Living, and Ageing in Halle
DBP: diastolic blood pressure
DM: diabetes mellitus
HL: health literacy
HLS-EU: European Health Literacy Survey
HLS-EU-Q16: HLS-EU-Questionnaire (short version)
HLS-EU-Q47: HLS-EU-Questionnaire (full version)
HRQL: health-related quality of life
OR: odds ratio
REALM: Rapid Estimate of Adult Literacy in Medicine
SBP: systolic blood pressure
SD: standard deviation
TOFHLA: Test of Functional Health Literacy in Adults

## References

[1] Nutbeam D (2000). Health literacy as a public health goal: a challenge for contemporary health education and communication strategies into the 21st century. Health Promot Int.

[2] Sørensen K, Van den Broucke S, Fullam J, Doyle G, Pelikan J, Słońska Z, et al. Health literacy and public health: a systematic review and integration of definitions and models. BMC Public Health. 2012;12(1):80. 10.1186/1471-2458-12-80 PMC3292515 22276600

[3] Berkman ND, Sheridan SL, Donahue KE, Halpern DJ, Crotty K (2011). Low health literacy and health outcomes: an updated systematic review. Ann Intern Med.

[4] Baker DW, Parker RM, Williams MV, Clark WS (1998). Health literacy and the risk of hospital admission. J Gen Intern Med.

[5] Parker RM, Baker DW, Williams MV, Nurss JR (1995). The test of functional health literacy in adults: a new instrument for measuring patients’ literacy skills. J Gen Intern Med.

[6] Weiss BD, Mays MZ, Martz W, Castro KM, DeWalt DA, Pignone MP (2005). Quick assessment of literacy in primary care: the newest vital sign. Ann Fam Med.

[7] Davis TC, Long SW, Jackson RH, Mayeaux EJ, George RB, Murphy PW (1993). Rapid estimate of adult literacy in medicine: a shortened screening instrument. Fam Med.

[8] HLS EU Consortium. The European Health Literacy Project (HLS-EU). 27-4-2012.

[9] Nutbeam D, Kickbusch I (2000). Advancing health literacy: a global challenge for the 21st century. Health Promot Int.

[10] Nutbeam D (2008). The evolving concept of health literacy. Soc Sci Med.

[11] Lee S-YD, Tsai T-I, Tsai Y-W, Kuo KN (2010). Health literacy, health status, and healthcare utilization of Taiwanese adults: results from a national survey. BMC Publ Health..

[12] Schipf S, Werner A, Tamayo T, Holle R, Schunk M, Maier W (2012). Regional differences in the prevalence of known Type 2 diabetes mellitus in 45–74 years old individuals: results from six population-based studies in Germany (DIAB-CORE Consortium). Diabet Med.

[13] Stang A, Döring A, Völzke H, Moebus S, Greiser KH, Werdan K (2011). Regional differences in body fat distributions among people with comparable body mass index: a comparison across six German population-based surveys. Eur J Cardiovasc Prev Rehabil.

[14] Greiser KH, Kluttig A, Schumann B, Kors JA, Swenne CA, Kuss O (2005). Cardiovascular disease, risk factors and heart rate variability in the elderly general population: design and objectives of the CARdiovascular disease, Living and Ageing in Halle (CARLA) Study. BMC Cardiovasc Disord..

[15] Greiser KH, Kluttig A, Schumann B, Swenne CA, Kors JA, Kuss O (2009). Cardiovascular diseases, risk factors and short-term heart rate variability in an elderly general population: the CARLA study 2002–2006. Eur J Epidemiol.

[16] Pelikan JM, Röthlin F, Canahl K. Introduction to HL measurement procedures of the HLS-EU study, 2nd European HL Conference, Aarhus, 10.4.2014. 2014.

[17] Adler N, Stewart J. The MacArthur Scale of subjective social status. In: Psychosocial Notebook. Psychosocial Working Group. 2007. http://www.macses.ucsf.edu/research/psychosocial/subjective.php. Accessed 07 Sep 2015.

[18] Ware JE, Kosinski M, Keller SD (1996). A 12-Item short-form health survey: construction of scales and preliminary tests of reliability and validity. Med Care.

[19] Cronbach LJ (1951). Coefficient alpha and the internal structure of tests. Psychometrika.

[20] Unterschiede bei der Gesundheitskompetenz Ergebnisse einer bundesweiten Repräsentativ-Umfrage unter gesetzlich Versicherten. WIdO-monitor 11(2), 1–12. 2014.

[21] Nakayama K, Osaka W, Togari T, Ishikawa H, Yonekura Y, Sekido A (2015). Comprehensive health literacy in Japan is lower than in Europe: a validated Japanese-language assessment of health literacy. BMC Public Health..

[22] van der Heide I, Rademakers J, Schipper M, Droomers M, Sørensen K, Uiters E (2013). Health literacy of Dutch adults: a cross sectional survey. BMC Public Health..

[23] Gazmararian JA, Baker DW, Williams MV, Parker RM, Scott TL, Green DC (1999). Health literacy among Medicare enrollees in a managed care organization. JAMA.

[24] Rothman RL, Housam R, Weiss H, Davis D, Gregory R, Gebretsadik T (2006). Patient understanding of food labels: the role of literacy and numeracy. Am J Prev Med.

[25] Barber MN, Staples M, Osborne RH, Clerehan R, Elder C, Buchbinder R (2009). Up to a quarter of the Australian population may have suboptimal health literacy depending upon the measurement tool: results from a population-based survey. Health Promot Int.

[26] Kobayashi LC, Wardle J, Wolf MS, von Wagner C (2014). Aging and functional health literacy: a systematic review and meta-analysis. J Gerontol B Psychol Sci Soc Sci.

[27] Paasche-Orlow MK, Parker RM, Gazmararian JA, Nielsen-Bohlman LT, Rudd RR (2005). The prevalence of limited health literacy. J Gen Intern Med.

[28] Toçi E, Burazeri G, Sørensen K, Kamberi H, Brand H (2015). Concurrent validation of two key health literacy instruments in a South Eastern European population. Eur J Public Health.

[29] Ricardo AC, Yang W, Lora CM, Gordon EJ, Diamantidis CJ, Ford V (2014). Limited health literacy is associated with low glomerular filtration in the Chronic Renal Insufficiency Cohort (CRIC) study. Clin Nephrol.

[30] Cunha M, Gaspar R, Fonseca S, Almeida D, Silva M, Nunes L (2014). Implications of literacy for health for body mass index. Aten Primaria..

[31] Schillinger D, Grumbach K, Piette J, Wang F, Osmond D, Daher C (2002). Association of health literacy with diabetes outcomes. JAMA.

[32] Cajita MI, Cajita TR, Han HR. Health literacy and heart failure: a systematic review. J Cardiovasc Nurs. 2015; Epub ahead of print. 10.1097/JCN.0000000000000229 PMC4577469 25569150

